# Supplementary material for: Transcriptomic Insights into Mechanisms of Early Seed Maturation in the Garden Pea (Pisum sativum L.)
Source: Cells. 2020 Mar 23;9(3):779. doi: 10.3390/cells9030779 (PMC7140803; doi:10.3390/cells9030779)

Sprint−2,20(3)

Global Summary

%DE = 0.23

genes with fdr < 0.2 = 475 (219 + / 256 −)

genes with fdr < 0.1 = 435 (207 + / 228 −)

genes with fdr < 0.05 = 415 (200 + / 215 −)

genes with fdr < 0.01 = 356 (170 + / 186 −)

genes in genesets = 0

<FC> = 0

<t−score> = 0

<p−value> = 0

<fdr> = 0.77

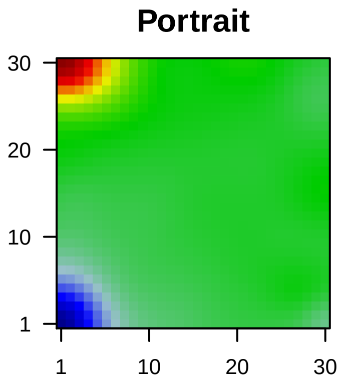

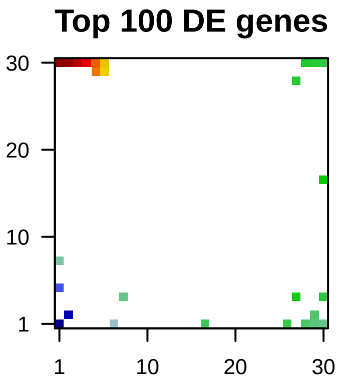

Global Genelist

Rank	ID	log(FC)	p−value	fdr	Metagene	Description
1	TRINITY_DN1	0.55	2e−16	2e−15	30 x 1	
2	TRINITY_DN2	−0.58	2e−16	2e−15	1 x 1	
3	TRINITY_DN9	−0.67	2e−16	2e−15	30 x 30	
4	TRINITY_DN7	−0.74	2e−16	2e−15	30 x 30	
5	TRINITY_DN5	−0.62	2e−16	2e−15	30 x 30	
6	TRINITY_DN5	−0.54	2e−16	2e−15	1 x 1	
7	TRINITY_DN7	−1.21	2e−16	2e−15	1 x 1	
8	TRINITY_DN9	1.54	2e−16	2e−15	4 x 30	
9	TRINITY_DN9	−0.73	2e−16	2e−15	30 x 1	
10	TRINITY_DN9	−0.39	2e−16	2e−15	30 x 30	
11	TRINITY_DN9	−0.81	2e−16	2e−15	30 x 30	
12	TRINITY_DN9	1.19	2e−16	2e−15	27 x 4	
13	TRINITY_DN2	2	2e−16	2e−15	1 x 30	
14	TRINITY_DN2	−0.67	2e−16	2e−15	1 x 1	
15	TRINITY_DN2	−0.76	2e−16	2e−15	30 x 1	
16	TRINITY_DN2	1.17	2e−16	2e−15	30 x 30	
17	TRINITY_DN2	0.42	2e−16	2e−15	1 x 30	
18	TRINITY_DN2	0.55	2e−16	2e−15	1 x 30	
19	TRINITY_DN2	0.44	2e−16	2e−15	6 x 29	
20	TRINITY_DN2	0.71	2e−16	2e−15	1 x 1	

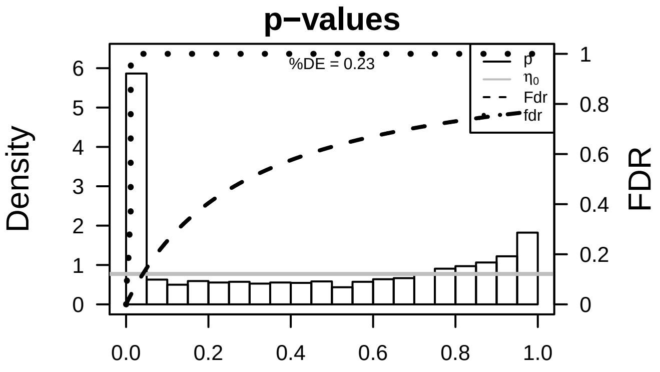

Supplement: Supplementary file 1 [file cells-09-00779-s001.zip › Supplementary materials/FileS4.zip!/FileS3/TFs - Results/Summary Sheets - Samples/Sprint.2.20.3..pdf]
